# Extracellular Vesicles: Multimodal Tools for Diagnosis, Prognosis, and Therapy in Respiratory Diseases

**DOI:** 10.1017/erm.2025.10025

**Published:** 2025-10-21

**Authors:** Miquéias Lopes-Pacheco

**Affiliations:** 1 Department of Pediatrics, Emory University School of Medicine, Atlanta, GA, USA; 2 Center for Cystic Fibrosis and Airways Disease Research, Emory University and Children’s Healthcare of Atlanta, Atlanta, GA, USA

**Keywords:** biomarker, inflammation, lung, mesenchymal stromal cell, miRNA, remodelling, therapeutic development

## Abstract

**Background:**

Respiratory diseases are increasing global health burden with persistently high morbidity and mortality. Extracellular vesicles (EVs), which are virtually released by all cell types and carry a variety of molecules like miRNAs, have emerged as crucial mediators of intercellular communication. They play a key role in maintaining lung homeostasis and are involved in the pathogenesis of various respiratory conditions. Furthermore, mesenchymal stromal cell-derived EVs (MSC-EVs) have shown significant therapeutic potential due to their anti-inflammatory, antimicrobial, and reparative properties.

**Methods:**

This narrative review critically assesses the current body of literature on the roles of EVs in respiratory diseases. We examine evidence from pre-clinical and clinical studies that investigate EVs as biomarkers and therapeutics for conditions including asthma, bronchopulmonary dysplasia (BPD), chronic obstructive pulmonary disease (COPD), cystic fibrosis (CF), idiopathic pulmonary fibrosis (IPF), lung cancer, and pulmonary arterial hypertension (PAH).

**Results:**

EVs reflect the physiological or pathological state of their parental cells, making them promising multimodal biomarkers for the early diagnosis and monitoring of disease progression. Additionally, MSC-EVs function as effective, cell-free therapeutic agents. In a variety of disease models, they demonstrate efficacy by modulating immune responses, enhancing alveolar fluid clearance, and restoring epithelial and endothelial barrier integrity, leading to improved survival and outcomes.

**Conclusions:**

EVs hold a dual and transformative potential in respiratory medicine. They may serve as valuable diagnostic and prognostic tools, and their application as cell-free therapeutics represents a novel and promising strategy for treating a wide spectrum of debilitating respiratory diseases.

## Introduction

Respiratory illnesses represent an increasing societal burden worldwide and encompass various pathological conditions, including obstructive and restrictive ventilatory disorders, pulmonary infections, and lung cancer. Among these, chronic respiratory diseases have a prevalence of more than 450 million cases and claim over four million lives annually, being therefore among the global leading causes of death (Refs. 1, 2). These diseases are characterized by chronic airway/alveolar inflammation and progressive deterioration of lung function, with common clinical symptoms including chronic cough, chest tightness, shortness of breath, and mucus production. Furthermore, pulmonary exacerbations can frequently occur and contribute to accelerating disease progression and increasing the mortality rate (Refs. 3–5). Despite considerable advances in understanding the underlying mechanisms in the pathogenesis and progression of severe respiratory diseases, there is an urgent need for more precise biomarkers and effective therapies to reduce morbidity and mortality associated with these devastating conditions.

**Figure 1.**
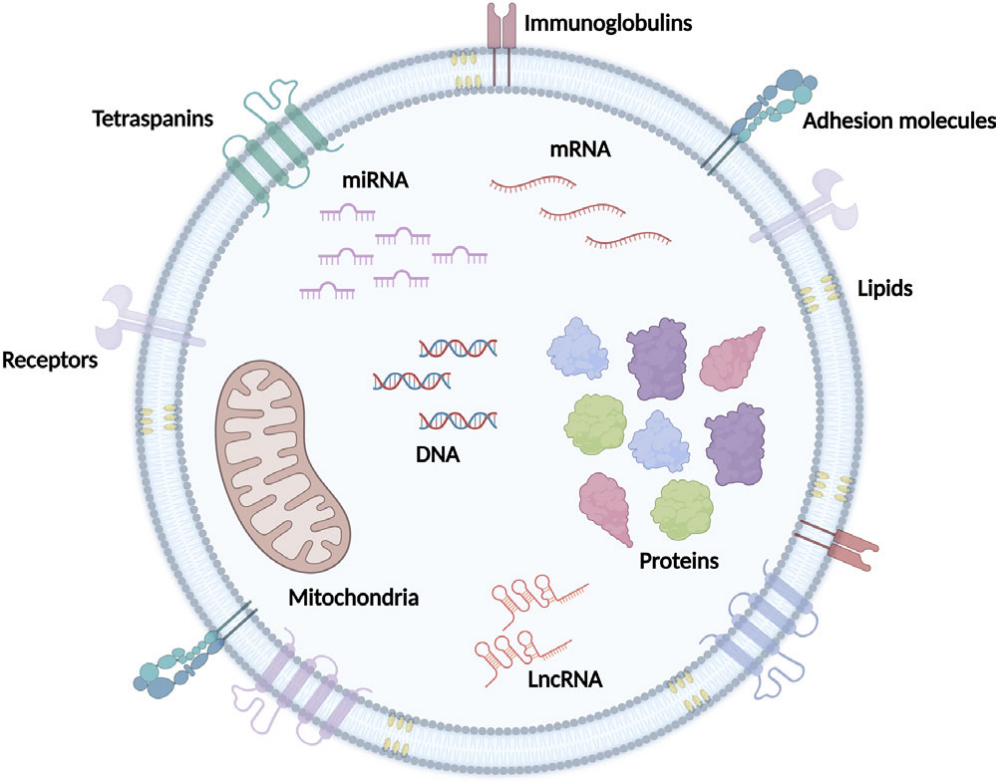
**Extracellular vesicle (EV) composition.** EVs are small, non-replicating membrane-bound particles released by cells into the extracellular environment. These particles are delimited by a lipid bilayer with membrane proteins (e.g., tetraspanins, receptors, immunoglobulins, and adhesion molecules), and their internal content is composed mainly of cytosolic proteins, lipids, and nucleic acids (e.g., DNA and RNA), which reflects the state of their parental cells. Some EVs can also contain metabolites and cellular organelles (e.g., mitochondria).

Extracellular vesicles (EVs) are a multifaceted group of non-replicating lipid bilayer membrane-delimited particles (Figure 1), which are ubiquitously released or shed by all cell types (Refs. 6–8). These particles can range from nano- to micrometres and once released into the extracellular milieu, they are extensively distributed and transported through biofluids (bloodstream, saliva, breast milk, urine, bronchoalveolar lavage fluid (BALF), and others), being thus able to travel long distances within the human body (Ref. 9). Accordingly, EVs play a key role in intercellular communication and participate in an intricate interplay among cells and tissues via autocrine, paracrine, or endocrine signalling mechanisms. Indeed, numerous studies have demonstrated that the internal content of EVs (nucleic acids, proteins, glycan structures, metabolites, and lipids) exhibits high stability due to protection from external enzymatic degradation (Refs. 10,11) and reflects the state of their cells of origin (Refs. 12–14). After binding to specific plasma membrane receptors (e.g., integrins, tetraspanin, proteoglycans, and immunoglobulin) of target cells, EVs activate ligand-receptor downstream signals or release their content into the cytosol (or deliver it to specific organelles) to promote alterations in gene and protein expression, signal transduction, cell proliferation, differentiation, and survival (Refs. 15, 16).

The first report potentially documenting the existence of EVs came in 1946 when Chargaff and West indicated the presence of ‘lipoproteins of very high particle weight’ in serum (Ref. 17). A subsequent study from Wolf in 1967 reported the acceleration of coagulation by lipid-rich particles originating from the granules of platelets (Ref. 18). In the 1980s, studies from Harding and Pan reported the fusion of multivesicular endosomes with the plasma membrane of rat and sheep reticulocytes and the subsequent release of their cargo into the extracellular space (Refs. 19, 20). Since then, a growing body of research has been performed to provide further understanding of EV biology from different cell types and their potential implications for diagnosis, prognosis, and therapeutics.

Due to large heterogeneity in the literature and aiming to guide EV studies, the International Society for Extracellular Vesicles (ISEV) has established minimal criteria for their definition and characterization (Refs. 6–8). Accordingly, EVs can be categorized based on their known cellular origin/biosynthesis, secretion pathway, and size (Table 1). Although significant progress has been attained in developing several methods for EV isolation and characterization (Tables 2 and 3), these are still unable to obtain pure EV populations since different EV types can overlap in size, density, and molecular composition. Furthermore, the identification of the cellular origin of EVs is particularly relevant in the clinical context, and certain markers are cell/tissue-specific; however, EV purification should not rely solely on EV-surface protein, and a limited consensus persists on appropriate markers to define biogenesis and origin for all EV subtypes.

**Table 1.** EV subtypes based on size, formation/origin, and related markers (Refs. 6–8)

EV subtype^a^	Size (nm)^b^	Other nomenclatures^c^	Formation/origin	Biomarker(s)
Small EVs (< 200 nm)	40–200	Exosomes	Originated from internal cellular compartments (endosomes and multivesicular bodies) and fuse with the plasma membrane to be released into the extracellular space through ESCRT-dependent or independent mechanisms	Alix, Hsp70, Tspans (CD9, CD63, CD81), Tsg101
	50–1000	Ectosomes, microvesicles, or microparticles	Directly budding outward from the plasma membrane	CD40, integrins, selectins, tissue factor
Large EVs (≥ 200 nm)				
	1000–5000	Apoptotic bodies	Cytoplasm fragmentation and release by cells undergoing apoptosis	Annexin A1, annexin V, C3b, histones, thrombospondin
	500–3000	Migrasomes	Produced by retraction fiber bifurcation during cell migration	Annexin A1, CD63, Tspan4
	>1000	Large oncosomes	Shed by oncocytes through plasma membrane budding and might share a biogenesis pathway with ectosomes	ARF6 or Cav–1, CK18

Abbreviations: Alix: ALIG-2-interacting protein X; ARF: ADP-ribosylation factor; Cav: caveolin; CK: cytokeratin; ESCRT: endosomal sorting complexes for transport; Hsp: heat shock protein; Tsg: tumor susceptibility gene; Tspan: tetraspanin.

a Recommended operational term based on the diameter of the separated particles.

b There is no strict consensus on size cut-offs since it may be affected by the separation method.

c Related to formation or specialized cell processes and recommended to be used only when subcellular origin can be demonstrated.

**Table 2.** Methods commonly used for EV separation and concentration

Technique/principle	Main advantage(s)	Main limitation(s)/drawback(s)	References
Differential (ultra)centrifugation: EV sedimentation based on increasing centrifugal forces	Easy to perform and most used method Suitable for multiple samples and/or large volumes	Time-consuming and may cause aggregation of EVs Obtaining full separation of EV subtypes is nearly impractical	(Refs. 222, 223)
Density gradient/cushion: EV separation into layers based on gradients is created using selected dense media	Allow for implementing different settings (e.g., bottom-up, top-down)	Low recovery of high-purity samples	(Refs. 223, 224)
Size exclusion: EV separation based on size using physical barriers	Easy to perform with well-defined kits available	Might require extensive post-isolation processing Limited sample volumes	(Refs. 225, 226)
Polymer precipitation: EVs are complexed for isolation by size or density	Efficient with commercially available kits	Relatively low purity Requires post-isolation decomplexation	(Refs. 39, 227)
Microfluidics: EV separation based on particle properties (acoustic, electric, magnetic, optical, or immunoisolation)	Easily automated and integrated with other principles for application in different fields High precision with qualitative potential	Limited throughput Requires specialized equipment	(Refs. 228, 229)
Affinity separation: EV separation based on their molecular composition or surface charge	High specificity and purity	More expensive and might be limited for certain EV subtypes	(Refs. 230, 231)

**Table 3.** Methods commonly used for EV characterization

Analytical approach	Assessment(s)	Standardized methods/techniques
Biophysical characterization	Analysis of size distribution, morphological structure, surface properties, and mechanical characteristics	Microscopy (electron microscopy, atomic force microscopy, and super-resolution optical microscopy) Spectroscopy (Fourier transform infrared spectroscopy and Raman spectroscopy) Scattering techniques (dynamic light scattering, nanoparticle tracking analysis, and small-angle X-ray scattering) Zeta potential measurements
Biochemical characterization	Identification of positive *versus* negative markers Analysis of protein, lipid, and nucleic acid content	Immunostaining (Western blot and immunofluorescence microscopy) Flow cytometry Mass spectrometry RNA sequencing, qPCR
Functional characterization	Analysis of their role in various biological processes and their potential as therapeutic agents	Fluorescent tracking assays Enzyme activity assays Cellular uptake and interaction assays *In vitro* and *in vivo* functional models (immune responses and tissue regeneration)

In the lungs, EVs have been demonstrated to be essential contributors to maintaining cellular homeostasis, regulating immune responses, and facilitating tissue healing/remodelling in both health and disease conditions (Refs. 13, 21). EVs promote these actions by operating as intercellular messengers and transferring their cargo to different lung resident and immune cells, which influence their behaviour and responses to environmental stimuli. In this review, we discuss the utility of EVs as biomarkers for early diagnosis and monitoring disease progression and their emerging role in therapeutic strategies for various respiratory diseases, including asthma, bronchopulmonary dysplasia (BPD), chronic obstructive pulmonary disease (COPD), cystic fibrosis (CF), idiopathic pulmonary fibrosis (IPF), lung cancer and pulmonary arterial hypertension (PAH).

## Influence of EVs in lung homeostasis and intercellular communication

**Figure 2.**
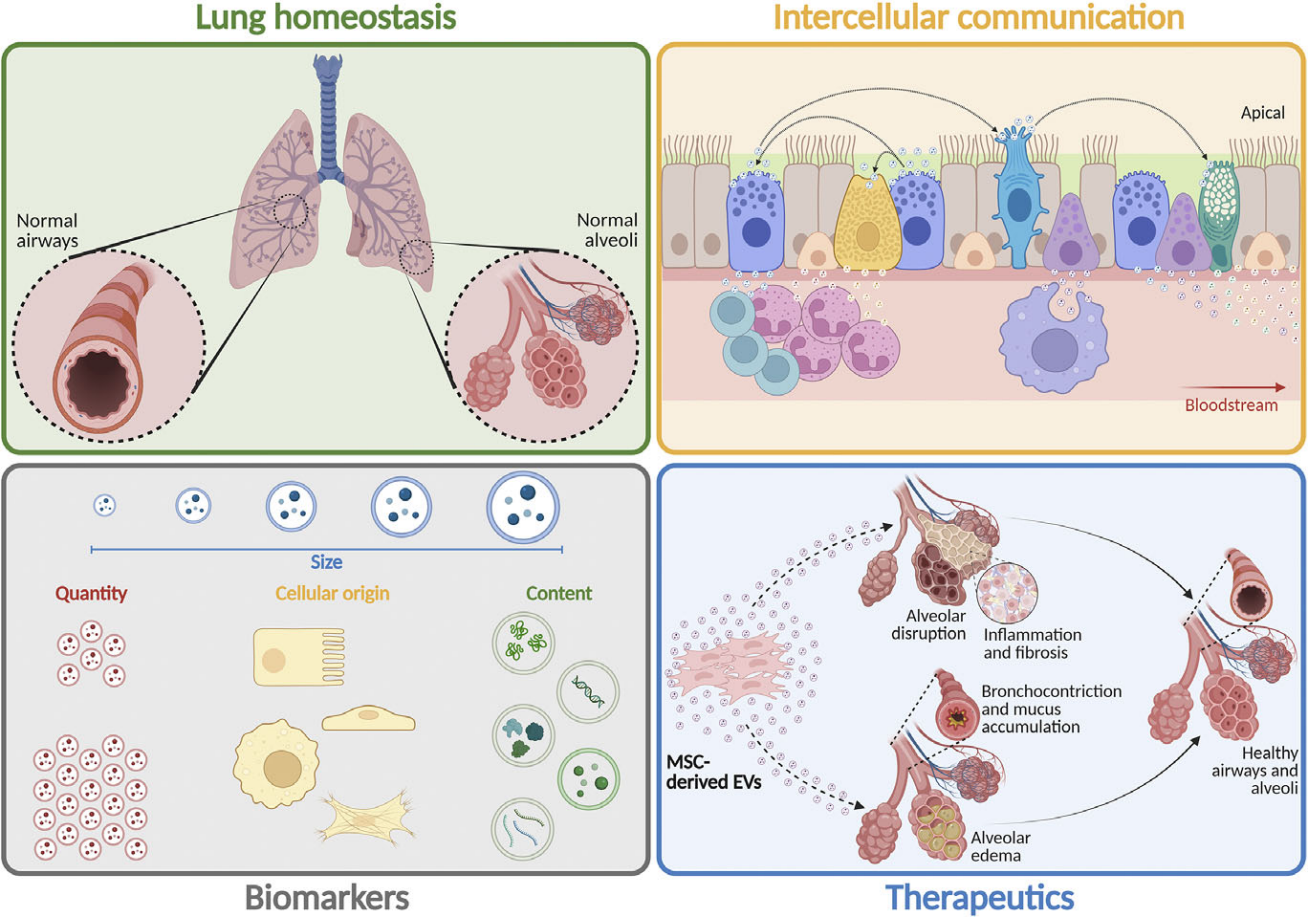
**Extracellular vesicles (EVs) in lung physiological or pathological processes.** The respiratory system is composed of diverse cell types, each with specialized roles, relying on intricate communication to preserve the structural and functional integrity of airways and alveoli. Under normal physiological (or pathological) conditions, EVs participate in lung homeostasis by promoting intercellular communication, thus regulating resident and immune cell responses, supporting barrier integrity, and facilitating tissue repair (or remodelling). EVs have been considered suitable biomarkers for diagnosis due to differences in their size, quantity, cellular origin, and content (both on the surface and within their lumen) in diverse diseases, which can facilitate the distinction between pathological conditions and severity with high accuracy and enable early and personalized therapeutic interventions. Mesenchymal stromal cell (MSC)-derived EVs have been demonstrated to be attractive therapeutic agents for respiratory diseases due to their ability to reduce inflammation and fibrosis, improve fluid clearance, and repair epithelial and endothelial barrier permeability.

The respiratory tract is composed of a variety of cell types with distinct functions that maintain their structural and functional integrity based on complex intercellular communication (Figure 2). Defence against invaders also requires well-coordinated communication between structural and immune cells to eliminate pathogens and prevent excessive inflammation that may lead to tissue damage and remodelling (Ref. 22). In this context, the airway epithelium acts as a physical barrier to prevent the entrance of allergens, pollutants, and pathogens and efficiently activates innate and adaptive immune responses for clearance and restoration of lung homeostasis (Ref. 23). EVs participate in such processes by supporting epithelial and endothelial cell function, enhancing barrier integrity, controlling oxidative stress, recruiting immune cells, and modulating their responses.

As bronchial epithelial cells are frequently exposed to pollutants and pathogens, their EVs contain factors that are essential for an innate mucosal defence, such as surface-associated mucin (MUC)-1, MUC-4, and MUC-16 (Refs. 24, 25). Bronchial epithelial cell-derived EVs are also a primary source of reactive pro-inflammatory mediators (Ref. 26) and demonstrated to activate receptor for advanced glycation end-products and mitogen-activated protein kinase pathways in neutrophils (Ref. 27). Meanwhile, pathogens defend themselves by releasing EVs with a cargo that prevents host immune responses and creates an optimal environment for their proliferation (Refs. 14, 28).

Alveolar epithelial cell-derived EVs exhibit abundant expression of mediators involved in the modulation of inflammatory signals (e.g., miR-223 and suppressor of cytokine signalling) (Refs. 29, 30). Endothelial cell-derived EVs assist in protecting lung tissue from inflammatory injury by delivering miR-10a and preventing monocyte activation (Ref. 31). Upon pulmonary artery damage, the abundance of EVs released by endothelial cells is significantly enhanced (Ref. 32). Endothelial cell-derived EVs can also participate in various biological processes, including angiogenesis, coagulation, and inflammation (Ref. 33).

Immune cell-secreted EVs play an important role in lung defence and homeostasis. Mast cell-derived EVs contribute to the production of antibody IgE (Ref. 34) and can stimulate epithelial-mesenchymal transition (EMT) of airway cells due to transforming growth factor (TGF)-β expression (Ref. 35). Immune cell-derived EVs can stimulate or inhibit fibroblast differentiation into myofibroblasts, contributing to tissue healing or remodelling (Ref. 36). Dendritic cell-derived EVs can affect T cell maturation and regulation (Ref. 34), while macrophage-derived EVs can promote the differentiation of monocytes by transferring miR-223 (Ref. 29). Altogether, these examples support the relevance of different cell type-derived EVs in protecting the respiratory tract against invaders and maintaining or restoring tissue homeostasis.

## EVs as biomarkers for respiratory diseases

Given the ability of EVs to carry molecular signatures that reflect the physiological or pathological state of their parental cells (Figure 2), they have been investigated as potential multimodal tools for disease diagnosis and prognosis as well as therapy monitoring (Refs. 12–14). Since EVs are abundantly present in all body fluids and have a relatively stable cargo, they represent a valuable source for biomarker assessment (Refs. 37, 38).

EVs have been frequently obtained from BALF and blood for respiratory research studies, with the latest representing a minimally invasive source that could be utilized for routine testing (Refs. 39, 40). EV profiling may also assist in identifying disease and monitoring progression, or even ensuring the differentiation of similar respiratory conditions, thus facilitating early and personalized interventions (Refs. 13, 41). For instance, alterations in the quantity and content of EVs have been documented in patients with asthma (Refs. 42, 43), COPD (Ref. 44), CF (Refs. 27, 43), and primary ciliary dyskinesia (Ref. 43). Analysis of EV-transported cargo was able to differentiate children with allergic airway disease from healthy controls (Ref. 45) as well as disease severity in COVID-19 (Refs. 40, 46).

### Asthma

Asthma is a complex respiratory disease characterized by chronic inflammatory responses that contribute to airflow obstruction and airway hyperresponsiveness, and remodelling (Ref. 47). Over 260 million people worldwide are affected by asthma with many individuals remaining undiagnosed and untreated (Ref. 1). Asthma has the highest prevalence among chronic respiratory diseases with variable incidence across different regions depending on several factors, including socioeconomic status and genetic predisposition (Ref. 48). EVs can be released by epithelial cells on both apical and basolateral sides (Refs. 49, 50), and these contribute to the pathogenesis and progression of asthma by influencing pro-inflammatory responses (Refs. 13, 51). For instance, house dust mite (HDM) extract significantly altered the content of BALF-derived EVs in experimental asthma (Ref. 52), corroborating findings of EVs from asthmatic patients (Ref. 53). BALF- and nasal lavage fluid-derived EVs from asthmatic patients exhibited increased concentrations of interleukin (IL)-4, leukotriene C4, and other chemoattractant factors (Refs. 54, 55). Likewise, an increase in EV release was observed in human bronchial epithelial cells treated with Th2 (IL-4 and IL-13) and Th17 cytokines (IL-17A and tumour necrosis factor (TNF)-α) (Ref. 56). Eosinophil-derived EVs from asthmatic patients were able to delay wound repair, promote *in vitro* apoptosis of small airway epithelial cells, and stimulate proliferation of smooth muscle cells (Refs. 57, 58). Treatment with EV-production inhibitor GW4869 ameliorated asthma symptoms in an animal model by reducing the release of lung cell-derived EVs (Ref. 52). Such reduction was also accompanied by a reduction in the number of inflammatory cells, bronchial hyperresponsiveness, and IgE levels (Ref. 59). In BALF samples, positive correlations were established for EV counts *versus* eosinophilia and EV counts *versus* IgE titer (Ref. 60). Profiling studies have been performed to further understand EV involvement in asthma-mediated inflammatory responses. In BALF-derived EVs, proteomic and lipidomic analyses revealed increased levels of toll-like receptor (TLR) signalling-related proteins (Ref. 43) and altered lipid mediator composition of EVs from asthmatic patients compared to healthy controls (Ref. 60).

Several studies have investigated EV-transported miRNA for potential biomarkers of asthma. Along these lines, EV miRNA content was significantly distinct in samples from asthmatic patients compared to healthy controls (Refs. 42, 61). In mice, HDM exposure altered the expression of >100 miRNAs in EV content, including miR-346 and miR-574-5p (Ref. 52). Most BALF-derived EVs are released from epithelial cells in experimental asthma; however, a significant increase in EV number from immune cells was evidenced following allergen exposure. Increased levels of miR-142a and miR-223 were found in immune cell-derived EVs from this animal model (Ref. 62). In plasma-derived EVs, miR-21 and miR-223 levels were increased in patients with moderate asthma compared to healthy controls (Refs. 63, 64). A promising miRNA to identify mildly asymptomatic asthma is let-7, which was demonstrated to be downregulated in BALF-derived EVs from patients with asthma (Ref. 61). On the contrary, PM2.5 led to asthma exacerbation by increasing levels of EV-packaged let-7i-5p in human bronchial epithelial cells (Ref. 65). PM2.5 also increased levels of EV-packaged miR-129-2-30 that led to increased secretion of inflammatory mediators (IL-6, IL-8, and TNF-α) by epithelial cells (Ref. 65). Levels of miR-126 were elevated in serum-derived EVs from patients with asthma and lung tissue of asthmatic mice (Ref. 66). Blood eosinophil counts positively correlated with miR-122-5a expression in plasma- and sputum-derived EVs with the potential to differentiate endotypes of asthma (Ref. 67). Compared to healthy controls, EVs from asthmatic epithelial cells demonstrated increased levels of miR-9, which has been associated with steroid-resistant neutrophilic asthma (Ref. 68).

### Bronchopulmonary dysplasia

BPD is a chronic lung disorder characterized by inflammation and disruption in airways and lung parenchymal vasculature, primarily affecting preterm infants or neonates requiring oxygen therapy. Although progress has been made in improving the survival of preterm infants, BPD remains a major cause of morbidity among survivors (Ref. 69). EVs have been isolated from various biofluids to investigate their role in BPD; however, studies have demonstrated that tracheal aspirate fluid is a feasible source for EV isolation and may represent the lung development stage in neonates or preterm infants (Refs. 70, 71). Indeed, gestational age was demonstrated to be directly and negatively correlated to EV concentration and size, respectively (Refs. 71, 72). Likewise, experimental hyperoxia-induced bronchial epithelial cells release more EVs of smaller size compared to cells under normoxia (Ref. 70). Umbilical cord-derived EVs from neonates who developed BPD reduced cell proliferation and capillary tube formation in cultured endothelial cells (Ref. 73). Differences in EV-surface markers have also been documented in neonates with BPD compared with those without it (Refs. 70, 71). Tracheal aspirate-derived EVs exhibited increased levels of CD14 and CD24 (usually expressed by immune and epithelial cells, respectively) when obtained from infants with BPD (Ref. 71). In experimental hyperoxia-induced BPD, rats demonstrated an increase in EV-packaged surfactant protein C in plasma. When these EVs were administered in control animals, lung and brain damage occurred, as well as morphological alterations consistent with BPD (Ref. 74).

Analysis of EV miRNAs for BPD has gained traction in recent years. Over 400 EV-derived miRNAs were differentially expressed in the umbilical cord of neonates with BPD compared to those without it (Ref. 73). Among these, the most significant reduction was observed for miRNA-103a-3p and miRNA-185-5p, while miRNA-200a-3p had increased expression (Ref. 73). An increase in endothelial cell proliferation and migration, and tube formation was observed by miR-103a-3p and miR-185-5p overexpression. On the contrary, miR-200a-3p overexpression prevented cellular angiogenic responses (Ref. 73). In another study, miRNA profiling revealed 40 miRNAs differentially expressed in EVs from infants with severe BPD compared to age-matched neonates without BPD (Ref. 70). The most sensitive alteration to predict BPD severity was the reduction of miR-876-3p. When exogenous miR-876-3p was administered in experimental hyperoxia-induced BPD, animals exhibited reduced inflammation and improved alveologenesis (Ref. 70). EV miR-21 has been implicated in adults with lung diseases and was upregulated in serum-derived EVs of preterm infants (≤32 weeks gestation) with *versus* without lung disorder (Ref. 75).

### Chronic obstructive pulmonary disease

COPD is a heterogeneous group of respiratory diseases characterized by progressive and partially irreversible restriction of airflow due to small airway damage (chronic bronchitis) or extensive alveolar wall disruption with airspace enlargement (emphysema) (Ref. 76). COPD has the highest mortality rate among chronic respiratory diseases (Ref. 1), with cigarette smoke (CS) representing the major risk factor for COPD development (Ref. 77). Alterations in lung structural and immune cell-derived EVs have been documented in COPD. Harmful gases or particles were demonstrated to stimulate the release of stressed lung cell-derived EVs that contribute to COPD-associated tissue damage (Ref. 78). Activated neutrophils participate in alveolar deterioration by enhancing the release of EV-transported elastase, which causes significant tissue damage by degrading the lung extracellular matrix (Ref. 79). An increase in the production of endothelial cell-derived EVs was also found in patients with exacerbated COPD (Ref. 44). Compared to patients with stable disease, exacerbated ones exhibited a greater number of serum-derived EVs, which correlated with IL-6, C-reactive protein, and soluble TNF-R1 values (Ref. 78). In an early study, a reduction in lung function (FEV_1_/FVC ratio) was correlated with increased release of endothelial cell-derived EVs in COPD (Ref. 44), while a subsequent study evidenced a negative correlation between FEV_1_ and the number of circulating EVs (Ref. 80). Because pathogens also release EVs, infected patients with COPD may present worse symptoms. For instance, *E. coli*-derived EVs could accelerate alveolar disruption by intensifying neutrophilic inflammation via IL-17A-dependent signalling (Ref. 81).

Mechanistic studies have provided novel insights into the onset and progression of COPD by investigating EV miRNA content. In human bronchial epithelial cells, CS extract exposure upregulated miR-21 and miR-210, increasing the production of α-smooth muscle actin and collagen type I and inducing lung fibroblast differentiation into myofibroblasts (Refs. 82, 83). These alterations facilitated tissue remodelling that was prevented by administering a miR-21 inhibitor (Ref. 83). CS-exposed bronchial epithelial cells also demonstrated upregulation of miR-500a-5p, miR-574-5p, miR-656-5p, miR-3180-5p, and miR-3913-5p, and downregulation of miR-130b-5p, miR-222-5p, and miR-618 (Ref. 84). Furthermore, EVs from CS-exposed endothelial cells were enriched with specific miRNAs (let-7d, miR-125a, miR-126, and miR-191) that, once engulfed by macrophages, inhibited their clearance abilities (Ref. 85). CS-exposed airway epithelial cells released EVs containing miR-7, miR-21-3p, miR-27b-3p, miR-125a-5p, and miR-221-3p that induced macrophage polarization toward pro-inflammatory M1 phenotype (Refs. 86–89). In another study using CS-induced emphysema, airway epithelial cells exhibited increased levels of EV miR-93, which increased levels of macrophage-produced metalloproteinases 9 and 12 and promoted lung remodelling by excessively degrading elastin (Ref. 90). CS-exposed airway epithelial cells can also contribute to lung remodelling by reducing EV-packaged miR-422a, leading to greater secretion of phosphoprotein-1 and production of collagen type I and smooth muscle actin by fibroblasts (Refs. 83, 91).

Several studies have investigated alterations in EV miRNA expression to distinguish patients with distinct severity of COPD or from other diseases. Levels of miR-199a-5p were upregulated in plasma-derived EVs from patients with COPD compared to non-smokers (Ref. 92). A negative correlation between this miRNA and FEV_1_ was observed (Ref. 93). In plasma-derived EVs, a set of four miRNAs (miR-92b-3p, miR-106b-3p, miR-223-3p, and miR-374a-5p) demonstrated high diagnostic accuracy in segregating stable and exacerbated patients (Ref. 94), while neutrophil count combined with serum-derived EV miR-1258 also demonstrated high accuracy for such (Ref. 95). Muscle dysfunction in COPD patients was detected by upregulation of three plasma-derived EV miRNAs (miR-133a-3p, miR-133a-5p, miR-206) (Ref. 96). Patients with lung cancer or stable COPD differed in the expression of 14 plasma-derived EV miRNAs, of which the highest diagnostic accuracy was found by the expression of miR-27a-3p combined with miR-106b-3p and miR-361-5p (Ref. 97). Inflammatory endotypes of COPD (neutrophilic *versus* eosinophilic) were also segregated by differentially expressed EV miRNAs in the BALF (Ref. 98).

### Cystic fibrosis

CF is a life-limiting genetic disease caused by mutations in the CF transmembrane conductance regulator (CFTR) gene and affects over 100,000 individuals worldwide. Despite the multiorgan involvement, CF morbidity and mortality primarily result from end-stage lung disease due to impaired mucociliary clearance, chronic inflammation, and recurrent infection (Ref. 99). Emerging evidence suggests that EVs participate in inflammation and immune responses in CF (Ref. 41). In this context, higher EV counts were observed in CF cell lines (CFBE41o^−^ and CuFi-5) compared to control cells (16HBE14o^−^ and Nuli-1) (Ref. 27). Differences in EV cargo from CF and non-CF cell lines were also evidenced in response to *Pseudomonas aeruginosa* (Ref. 100), which is the most prevalent pathogen found in CF lungs. Interestingly, primary bronchial epithelial cell-derived EVs inhibited *P. aeruginosa* biofilm formation by mitigating levels of biofilm-related proteins in a CF mouse model (Ref. 101). On the contrary, both WT and CF airway epithelial cell-derived EVs exposed to *P. aeruginosa* exhibited greater content of pro-inflammatory mediators; however, responses from CF macrophages were mitigated in comparison to WT macrophages (Ref. 102). BALF-derived EVs revealed an enrichment of protein signatures related to macrophage activation and inflammation in a murine model of CF-like muco-inflammatory lung disease (Ref. 103).

In sputum from CF patients, a high number of EVs – predominantly from granulocytes – was evidenced, and when these EVs were intratracheally administered in mice, there was strong neutrophilia in their lungs (Ref. 104). Likewise, CF BALF-derived EVs contained higher levels of leukocyte chemotaxis-related proteins and drove neutrophil recruitment (Refs. 27, 43). By analysing BALF-derived EVs, unique protein signatures were identified to differentiate CF stable and exacerbated *versus* controls (Ref. 27). BALF-derived EVs from paediatric CF patients (similar to those from asthmatic patients) were found to enhance epithelial sodium channel activity in small airway epithelia (Ref. 105). Compared to controls, CF EVs exhibited alterations in lipid profile with increased ceramide production, which might accelerate the release of EV-packaged pro-inflammatory ceramides and perpetuate the inflammatory state in CF (Ref. 106). CF airway fluid-derived EVs demonstrated an enrichment with IL-1α, IL-1β, IL-18, and active caspase-1, which stimulate a hyperinflammatory state by inducing inflammasome activation of surrounding cells (namely, epithelial cells and newly recruited neutrophils) (Ref. 107). In sputum-derived EVs from CF patients treated for pulmonary exacerbation, levels of EV-transported neutrophil elastase and myeloperoxidase were decreased and demonstrated good correlation with improvement in lung function (Ref. 108).

While BALF and sputum have been utilized as the main sources of EVs in CF studies, recent evidence demonstrated that urine-derived EVs can be a suitable source to distinguish CF patients and healthy controls (Ref. 109). Indeed, CF EVs exhibited increased expression of epidermal growth factor receptor (EGFR) and decreased expression of klotho and matrisome, providing insights into CFTR-related kidney dysfunction (Ref. 109).

Recent studies have assessed the impact of clinically approved CFTR modulators on CF EVs (Refs. 27, 110) since these drugs can correct the basic defects of the mutant CFTR protein (Refs. 99, 111). In CFBE41o^−^ cells, treatment with lumacaftor/ivacaftor or tezacaftor/ivacaftor led to a significant reduction of EV release (Ref. 27). Although children with CF and healthy controls demonstrated equivalent EV counts, treatment with elexacaftor/tezacaftor/ivacaftor altered EV protein content in serum-derived EVs of patients with CF from different age groups (Ref. 110).

### Idiopathic pulmonary fibrosis

IPF is a progressive disorder characterized by abnormal deposition of extracellular matrix in diverse regions of the lung parenchyma in response to chronic epithelial cell damage. The disease affects over three million people worldwide and presents a median survival rate of 2–5 years with only 20–25% of patients living beyond 10 years (Ref. 112). Under normal conditions, lung fibroblasts communicate with surrounding cells by EVs enriched with prostaglandins to inhibit myofibroblast differentiation; however, a reduction in EV-transported prostaglandins could trigger pulmonary fibrosis (Refs. 36, 113). Several studies have demonstrated the detrimental influence of EV-transported cargo on cell senescence and EMT during IPF development and progression. For instance, decreased reparative properties were observed for senescent cell-derived EVs (Refs. 114, 115). Administration of syndecan-1-positive-EVs into mice lungs led to epithelial reprogramming by upregulating TGF-β and WNT signalling (Ref. 116). WNT signalling plays a role in IPF pathogenesis by promoting fibroblast proliferation, and an elevated number of WNT5A-positive EVs was found in BALF from IPF patients compared to non-IPF controls (Ref. 117). Fibroblast-derived EVs contain high levels of fibronectin on their surface that, once bound to other fibroblasts, stimulate invasion by focal adhesion kinase- and steroid receptor coactivator kinase-mediated mechanisms (Ref. 118).

Various studies have investigated the influence of EV-transferred miRNAs on aberrant EMT and fibrosis development. For instance, bronchial epithelial cells from healthy subjects release much less EVs than those cells from IPF patients, which also contain increased levels of miR-7, miR-137, miR-195, and miR-411. These EVs can also induce senescence in naïve epithelial cells (Ref. 119). In IPF sputum-derived EVs, seven miRNAs were upregulated, and there was a negative correlation between miR-142-3p levels and lung diffusing capacity (Ref. 120). Interestingly, increased expression of miR-142-3p was suggested to come from macrophages and was able to reduce TGFβ-R1 expression in lung fibroblasts and alveolar epithelial cells (Ref. 121). Compared to non-IPF cells, lung fibroblasts from IPF patients release more EVs that can induce increased senescence and mitochondrial dysfunction in airway epithelial cells (Refs. 113, 118). IPF fibroblast-derived EVs carry high levels of miR-23b-3p and miR-494-3p, which lead to epithelial phenotypic alterations by inhibiting sirtuin-3. Such mechanisms may be involved in mitochondrial dysfunction and increased levels of reactive oxygen species that result in DNA damage and epithelial cell senescence (Ref. 113). Upregulation of miR-21-5 was found in serum-derived EVs of IPF patients with a good prediction of mortality in the 30-month follow-up (Ref. 122). Levels of EV-transferred miR-21-5p and the rate of decline of spirometry FVC also demonstrated good correlation (Ref. 122).

### Lung cancer

Lung cancer is the leading cause of malignant neoplasms and accounts for ~25% of all global cancer deaths (Ref. 123). Extensive research has been performed to investigate EVs and their cargo for early cancer detection. For instance, increased expression of EGFR has been detected in lung cancer cells with an even higher level of EGFR in EVs from patients with lung cancer compared to healthy controls (Ref. 124). Lung cancer cell-derived EVs contain miR-21 and miR-29a that bind to TLR8 on immune cells within the tumour niche, leading to a pro-inflammatory state via NF-κB activation that results in tumour progression and metastasis (Ref. 125). Early tumour formation is usually marked by hypoxia with increased levels of EV hypoxic signature proteins associated with EMT (Ref. 126). Under hypoxic conditions, lung cancer cell-derived EVs express high levels of EGFR, TGF-β, miR-23a, and miR-619-5p that create an immunosuppressive state by polarizing macrophages (Ref. 127) and eliminating NK cells (Ref. 128), while inducing angiogenesis (Ref. 125), thus facilitating the dissemination of abnormal cells.

The cargo of lung cancer-derived EVs contributes to drug resistance (Ref. 129). Analysis of lung cancer-derived EVs indicated cisplatin response linked to high expression of miR-146a-5p and cisplatin resistance associated with miR-96 and miR-425-3p (Refs. 124, 130). Furthermore, poor prognosis was observed by miR-378 upregulation of serum-derived EVs from patients with non-small cell lung cancer (NSCLC) (Ref. 130). In patients with lung adenocarcinoma, plasma EVs exhibited elevated expression of Ras homolog family member V. By EV array, CD151/tetraspanin-24, CD171/L1CAM, and tetraspanin-8 were found to be abnormally expressed in histological slices of lung tumours (Ref. 131). Prospective miRNAs for early diagnosis and prognosis have been identified for lung cancer. In transcriptomic analysis, miR-30a-3p, miR-30e-3p, miR-181-5p, and miR-361-5p were identified for the diagnosis of lung adenocarcinoma, while miR-10b-5p, miR-15b-5p, and miR-320b were suggested for lung squamous cell carcinoma (Ref. 132). Prediction of survival rate by lung cancer was also suggested by analysis of let-7, miR-137, miR-182, miR-221, and miR-372 expressed in lung cancer cell-derived EVs (Ref. 132).

### Pulmonary arterial hypertension

PAH is a rare, progressive disease characterized by pulmonary vascular inflammation and remodelling that leads to right ventricular failure. In PAH patients, the number of circulating EVs was demonstrated to be significantly increased (Refs. 133–135) and directly correlated with functional impairment (Ref. 133) and mortality rate (Ref. 136). Proteomic analysis revealed 13 proteins associated with coagulation and oxidative stress that were differentially expressed in blood-derived EVs from PAH patients compared to healthy controls (Ref. 135). Endothelial cell-derived EV counts and levels of tricuspid annular plane systolic excursion (prognostic predictor for hypertension) were also higher in urine samples from PAH patients compared to controls (Ref. 137). Treatment with prostacyclin analogues reduced platelet- and leukocyte-derived EV counts and inhibited platelet reactivity and thrombus formation in PAH patients (Ref. 138).

EV content has been profiled to identify miRNAs and other factors that may be useful for PAH diagnosis and prognosis. For instance, miR-26a-5p was downregulated and miR-486-5p was upregulated in plasma-derived EVs of PAH patients (Ref. 139). A high level of miR-596 in plasma-derived EVs was associated with severe PAH and poor prognosis (Ref. 140). Translationally controlled tumour protein has increased expression in lung tissue sections of PAH patients, and its transfer by EVs led to proliferation and apoptosis resistance of pulmonary arterial smooth muscle cells that resulted in vascular remodelling (Ref. 141). In experimental monocrotaline-induced PAH, EV-packaged miR-211 induced proliferation of pulmonary arterial smooth muscle cells by inhibiting CaMK1/PPAR-γ signalling. Downregulation of miR-211 mitigated PAH in rats (Ref. 142).

### Other respiratory diseases

Recent evidence indicates that cilia-derived EVs possess distinct mechanisms of release and molecular features compared to cytosolic-derived EVs (Ref. 143). In primary ciliary dyskinesia, a rare genetic disease caused by mutations in genes related to cilia development and function, proteomic analysis demonstrated high levels of proteins involved in leukocyte chemotaxis and antioxidant activity (Ref. 43).

Both silicosis and pulmonary sarcoidosis exhibit granuloma formation and extensive lung parenchyma fibrosis resulting from different aetiologies. While silicosis is caused by silica inhalation mainly in work environments, systemic inflammation with unknown causes is responsible for sarcoidosis. In experimental silicosis, macrophage-derived EVs contributed to silica-induced lung fibrosis by activating fibroblasts in a mechanism dependent on endoplasmic reticulum stress. A significant decrease in lung fibrosis and levels of pro-inflammatory mediators (TNF-α, IL-1β, and IL-6) in BALF was observed when animals were pre-treated with the EV-production inhibitor GW4869 (Ref. 144). Serum-derived EVs from an animal model of silicosis demonstrated increased levels of miR-107 and miR-125-5p, which can promote fibroblast differentiation and contribute to lung fibrosis (Refs. 145,146). In addition, levels of miR-223-3p were significantly altered in EVs and tissue from silicosis patients, thus being considered a promising biomarker (Ref. 147). On the contrary, sarcoidosis patients demonstrated altered levels of CD14 and lipopolysaccharide-binding protein on serum-derived EVs by proteomics analysis (Ref. 148). Treatment with methotrexate also reduced levels of EV-transported serpin C1 in sarcoidosis patients, suggesting that it can be a good predictor for therapy monitoring (Ref. 149).

## Emerging therapeutic properties of EVs for respiratory diseases

**Figure 3.**
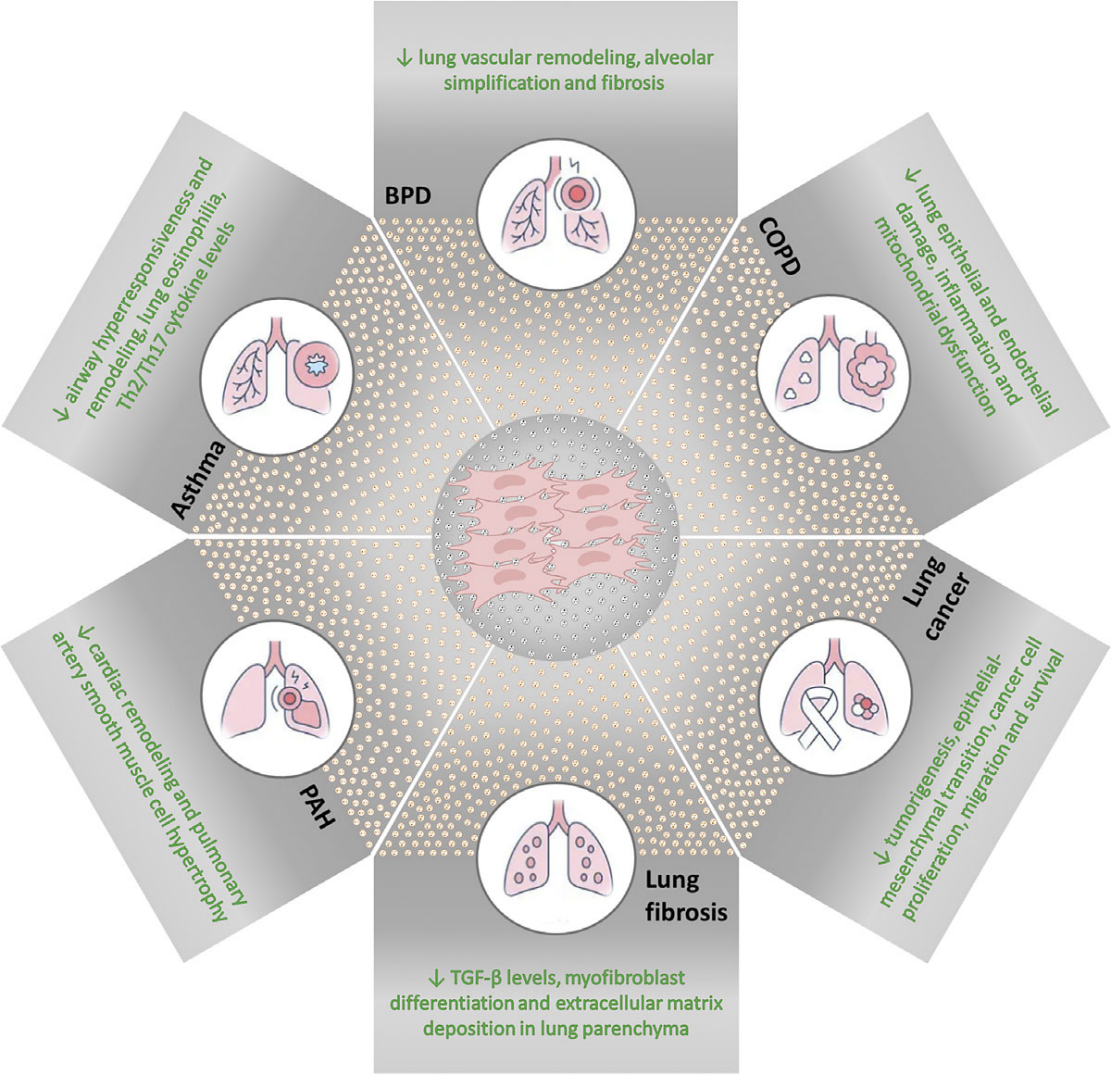
**Mesenchymal stromal cell (MSC)-derived extracellular vesicles (EVs) in respiratory diseases.** Major therapeutic effects of MSC-derived EVs were found in experimental models of asthma, bronchopulmonary dysplasia (BPD), chronic obstructive pulmonary disease (COPD), lung cancer, lung fibrosis, and pulmonary arterial hypertension (PAH).

One of the most attractive applications of EVs is in cell-free therapeutics, as these particles carry several molecules that can modulate lung resident and immune responses and assist in tissue repair (Figure 2). In this context, mesenchymal stromal cell (MSC)-derived EVs are particularly promising for such purposes due to their multimodal ability to promote anti-inflammatory and antimicrobial actions, fluid clearance, and recovery of epithelial and endothelial permeability in a variety of experimental models of respiratory diseases (Figure 3). As EVs are non-replicating, they are also considered safer and easier to store and distribute for therapeutic purposes than living cells. In addition, recent evidence indicates that EV therapeutic properties may be potentiated by stimulating MSCs with biological, chemical, or physical methods before EV isolation (Ref. 150).

### Asthma

Therapeutic effects of MSC-derived EVs have been investigated in several experimental models of asthma, including *Aspergillus* hyphal extract, HDM, and ovalbumin. In an early study, MSC-derived conditioned medium prevented peribronchial inflammation and airway smooth muscle thickening in experimental asthma by an adiponectin-promoted mechanism (Ref. 151). Subsequent studies demonstrated that EVs from both murine and human MSCs reduce airway hyperresponsiveness and lung inflammation by mitigating BALF neutrophil and eosinophil counts and levels of IL-4, IL-5, and IL-17 in *Aspergillus* hyphal extract-induced asthma (Ref. 152). MSC-derived EVs also stimulated T regulatory cell proliferation and immunosuppressive properties by enhancing IL-10 and TGF-β levels in peripheral blood mononuclear cells of asthmatic patients (Ref. 153).

In experimental ovalbumin-induced asthma, MSC-derived EVs were efficient at reducing lung eosinophilia, fibrosis, and IgE and TGF-β levels (Refs. 154, 155), decreasing the number of mucus-producing goblet cells (Ref. 156) and modulating macrophage polarization (Ref. 157). MSC-derived EVs mitigated airway remodelling by promoting M2-like macrophage activation via FoxO1 signalling (Ref. 158). TNF receptor-associated factor 1 was also involved in macrophage polarization by MSC-derived EVs by regulating NF-κB and AKT signalling pathways (Ref. 159). Furthermore, EMT and airway remodelling in asthma are influenced by WNT/β-catenin signalling activation, and MSC-derived EVs prevented tissue remodelling by inhibiting WNT/β-catenin signalling pathway-related factors. Such effects were reversed by the administration of BML-284, a WNT agonist (Ref. 156). Likewise, the downregulation of miR-188, an EV-enriched miRNA, mitigated the protective effects of MSC-derived EVs in experimental ovalbumin-induced asthma (Ref. 160). EV-transferred miR-221-3p inhibited FGF2 expression and ERK1/2 signalling, leading to mitigated lung inflammation and remodelling in experimental asthma (Ref. 161). In samples from asthmatic patients, STAT3 and miR-301a-3p levels were demonstrated to be negatively correlated. MSC-derived EV miR-301a-3p was efficiently internalized by airway smooth muscle cells, which inhibits their proliferation and migration by targeting STAT3 (Ref. 162). miR-223-3p is highly expressed in MSC-derived EVs and has a major protective action on asthma-induced airway remodelling by regulating NLRP3/caspase-1/gasdermin D-mediated inflammasome activation and pyroptosis (Ref. 163).

Under hypoxic conditions, a higher number of EVs was released by MSCs (Refs. 164, 165). When these EVs were administered in ovalbumin-induced asthma, there was greater improvement in lung inflammation (BALF total cell and eosinophil counts and levels of IL-4 and IL-13) and remodelling (levels of α-smooth muscle actin, collagen-1, and TGF-β1) compared to EVs from normoxia-cultured MSCs (Ref. 164). Nebulization of hypoxic MSC-derived EVs was also effective at reducing lung inflammation and remodelling (Ref. 166), levels of IgE and pro-inflammatory cytokines (Ref. 167). Upregulation of miR-146a-5p was evidenced on these EVs, and reduction of this EV-transferred miRNA impaired lung protective effects in experimental ovalbumin-induced asthma (Refs. 164, 166).

### Bronchopulmonary dysplasia

Several studies have investigated the potential of MSC-derived EVs for BPD therapy. In an initial study, MSC-derived EVs were demonstrated to improve pulmonary hypertension and lung function by reducing vascular remodelling, alveolar simplification, and fibrosis in hyperoxia-induced BPD in newborn mice (Ref. 168). These effects were associated with macrophage polarization to M2 anti-inflammatory phenotype instead of M1 pro-inflammatory one (Ref. 168). Both early and late administration of MSC-derived EVs were effective at improving core features of BPD on this model, thus preventing or reversing cardiorespiratory abnormalities (Ref. 169). Biodistribution analysis revealed MSC-derived EVs in lung tissue of hyperoxia-exposed newborn mice and epigenetic and phenotypic reprogramming of myeloid cells to a non-inflammatory phenotype (Ref. 170). MSC-derived EVs also restored thymocyte development/maturation profile and thymic medullary structure by enhancing the expression of antioxidant-stress-related genes (Ref. 171).

In a rat model of hyperoxia-induced BPD, MSC-derived EVs reduced alveolar simplification, lung damage, and fibrosis in a dose-dependent manner (Ref. 172). *In vitro* analysis revealed that EVs delay the transdifferentiation of alveolar type II cells into type I cells by downregulating WNT5a (Ref. 172). Antenatal administration of MSC-derived EVs was also able to reduce NLRP3- and IL-1β-mediated inflammation and preserve distal lung growth and mechanics in chorioamnionitis-induced rat BPD (Ref. 173). These therapeutic benefits were associated with increased expression of vascular endothelial growth factor (VEGF) (Ref. 173), which is decreased in preterm infants (Ref. 174). Along these lines, EV-transferred VEGF mitigated lung injury by decreasing inflammation and improving alveolarization and angiogenesis in hyperoxia-induced rat BPD. Such effects were not found by EVs from VEGF-knockdown MSCs (Ref. 175). Neurologic alterations are also observed in infants with BPD. MSC-derived EVs not only reduced lung damage but also improved neurodevelopmental features in an animal model (Ref. 176).

### Chronic obstructive pulmonary disease

Initial studies assessed the effects of MSC-derived conditioned media in COPD models and demonstrated a variety of therapeutic benefits. In CS-induced emphysema, conditioned media promoted tissue repair and increased the number of small pulmonary vessels (Ref. 177). Hepatocyte growth factor-mediated protection was observed in lung tissue when MSC-derived conditioned media were administered at the onset of elastase-induced emphysema (Ref. 178).

EVs released by damaged alveolar type II cells promoted MSC migration and upregulated genes related to mitochondrial synthesis and transfer (Ref. 179). In co-culture experiments, MSCs released EVs to protect the BEAS2B lung epithelial cell line against CS-induced mitochondrial abnormalities (Ref. 180). MSC-derived EVs also reduced CS-induced mitochondrial dysfunction, peribronchial and perivascular inflammation, alveolar septa thickening, and mucus-producing goblet cell counts in rodent lungs (Refs. 180–182). CS exposure elevated pyroptosis and inhibited phagocytic abilities in alveolar macrophages, which was reversed by MSC-derived EVs (Ref. 182). In experimental papain-induced emphysema, MSC-derived EVs prevented endothelial cell apoptosis by activating VEGF/VEGF receptor-2-mediated AKT and MEK/ERK signalling pathways (Ref. 183). MSC-derived EVs were also able to improve lung function and mitigate airway inflammation and levels of pro-inflammatory cytokines in CS-exposed mice (Ref. 184). Interestingly, cardiorespiratory abnormalities in emphysematous mice were mitigated by MSC-derived EVs from healthy donors but not from elastase-induced emphysema (Ref. 115). These EVs exhibited downregulation of various anti-inflammatory and anti-oxidant mediators (Ref. 115). Artificial EVs from MSCs were generated by sequential penetration through polycarbonate membranes and demonstrated similar size, shape, and surface markers as natural MSC-derived EVs. When these EVs were administered in a model of elastase-induced emphysema, lower doses of artificial EVs promoted similar reparative actions as higher doses of natural EVs (Ref. 185).

### Lung cancer

Current research suggests MSCs as a double-edged sword in cancer as they demonstrate pro- and anti-tumorigenic actions (Refs. 186, 187). On the contrary, most studies revealed tumour-suppression effects by MSC-derived EVs, although further research is still needed. MSC EV-overexpressing miR-30b-5p prevented tumorigenesis and promoted NSCLC cell apoptosis by regulating the EZH2/PI3K/AKT pathway (Ref. 188). Both miR-204 and miR-598 have reduced expression in NSCLC tissues, which was associated with poor prognosis (Refs. 189, 190). MSC-derived EVs transferred miR-598 into NSCLC cells and prevented proliferation and migration by inhibiting THBS2 production (Ref. 189). Inhibition of EMT and NSCLC cell migration and invasion was also evidenced by EV-transferred miR-204 by targeting the KLF7/AKT/HIF-1α pathway (Ref. 190). NSCLC tissues exhibited high expression of CCNE1 and CCNE2 and low expression of miR-144. MSC-derived EVs reduced NSCLC proliferation and the number of S-phase-blocked cells by transferring miR-144 in both *in vitro* and *in vivo* models (Ref. 191). Another therapeutically relevant miRNA in lung cancer is miR-320a which, once transferred by MSC-derived EVs, reduced cancer cell migration and invasion by binding to sex-determining region Y-box 4 (Ref. 192). Let-7i has a reduced expression in lung cells, and its transfer by MSC-derived EVs inhibited lung cell proliferation by downregulating the KDM3A/DCLK1/FXYD3 signalling pathway (Ref. 193).

Despite promising therapeutic actions, some studies demonstrated an opposite effect in which MSC-derived EVs facilitate lung cancer development. In lung adenocarcinoma cells, EV-transferred miR-410 increased their proliferation and reduced apoptosis (Ref. 194). Under hypoxic conditions, MSC-derived EVs express certain miRNAs (miR-21-5p, miR-193a-3p, miR-210-3p, and miR-5100) that activate STAT3-mediated EMT and accelerate cancer cell invasion (Refs. 195, 196).

### Lung fibrosis

MSC-derived EVs have demonstrated a variety of therapeutic benefits in experimental lung fibrosis. In lung fibroblasts, MSC-derived EVs inhibited TGF-β1-mediated myofibroblast differentiation by a Thy-1-dependent mechanism (Ref. 197). Blockage of Thy-1 reduced EV uptake and preserved myofibroblast differentiation (Ref. 197). MSC-derived EVs mitigated bleomycin-induced lung fibrosis by stimulating the proliferation of bronchoalveolar stem cells (Ref. 198). Proteomic analysis demonstrated that human MSC-derived EVs modulate macrophages to an anti-inflammatory phenotype, thus mitigating lung inflammation and fibrosis in a model of bleomycin-induced lung fibrosis (Ref. 199). In PM2.5-induced lung fibrosis, MSC-derived EVs reduced levels of reactive oxygen species and apoptosis of alveolar epithelial cells (Ref. 200). MSC-derived EVs were effective at mitigating macrophage counts, collagen fibre content, and granuloma size in lung parenchyma, thus improving lung function in silica-induced lung fibrosis (Ref. 201). EVs from three-dimensional cultured MSCs also reduced collagen deposition in silica-exposed fibroblasts (Ref. 202).

The influence of MSC EV-transferred miRNAs in preventing or reversing lung fibrosis has been documented in several studies. For instance, miR-29b-3p demonstrated low expression in lung tissue, and its overexpression in MSC-derived EVs prevented interstitial fibroblast proliferation, differentiation, migration, and invasion by targeting frizzled 6 (Ref. 203). EV-transferred miR-186 also prevented fibroblast activation by downregulating SRY-related HMG box transcription factor 4 and its downstream gene Dickkopf-1, thus alleviating lung fibrosis in mice (Ref. 204). TGF-β signalling pathway was inhibited by EV-transferred miR-21 and miR-23, preventing myofibroblast differentiation and lung damage in experimental bleomycin-induced lung fibrosis (Ref. 205). EV-transferred let-7 mitigated lung epithelial fibrosis and remodelling in mice by reducing NLRP3-mediated inflammation activation, mitochondrial damage, and levels of reactive oxygen species in alveolar epithelial cells (Ref. 206). Levels of miR-30b were downregulated in the serum of IPF patients, and EV-transferred miR-30b reduced inflammation and apoptosis of TGF-β1-stimulated lung epithelial cell lines by targeting Runx1 and Spred2 (Ref. 207). EV-transferred miR-29c and miR-129 reduced lung damage and myofibroblast differentiation in experimental bleomycin-induced lung fibrosis (Ref. 208). In radiation-induced pulmonary fibrosis, EV-transferred miR-214-3p reduced endothelial cell damage, inflammation, and fibrosis by downregulating ataxia telangiectasia mutated/p53/p21-mediated signalling pathway (Ref. 209). EV-transferred miR-218 inhibited endothelial-mesenchymal transition and restored endothelial properties by downregulating the MeCP2/BMP2 pathway (Ref. 210). Knockdown of miR-218 reduced the therapeutic effects of MSC-derived EVs on endothelial-mesenchymal transition (Ref. 210).

In silica-induced lung fibrosis, MSCs release EV-containing miRNAs that inhibit macrophage activation by suppressing TLR signalling (Ref. 211). EV-transferred miR-223-3p reduced silica-induced inflammation (IL-1β, IL-18, and cleaved caspase-1) and remodelling (collagen I and III, fibronectin, and α-smooth muscle actin) by suppressing NLRP3-mediated signalling pathway (Ref. 147). In experimental silicosis, EV-containing let-7i-5p prevented fibroblast activation by targeting the TGF-β receptor/Smad3 pathway (Ref. 212), while EV-transferred let-7d-5p reduced PM2.5-induced lung fibrosis (Ref. 200). Likewise, miR-26a-5p is downregulated in silicosis, and its transfer by MSC-derived EVs prevented EMT by targeting Adam17/Notch signalling, thus mitigating lung fibrosis in mice (Ref. 213).

### Pulmonary arterial hypertension

In early studies, MSC-derived conditioned media demonstrated protective effects against PAH by preventing cardiac remodelling and improving pulmonary blood flow (Ref. 214) and by downregulating calcineurin and nuclear factor of activated T-cells, thus inhibiting the inflammation-mediated over-proliferation of pulmonary artery smooth muscle cells (Ref. 215). Subsequently, MSC-derived EVs reduced right ventricular hypertrophy and small pulmonary artery area index in experimental sugen/hypoxia- (Ref. 216) and monocrotaline-induced PAH (Ref. 217). MSC-derived EVs promoted therapeutic effects on experimental monocrotaline-induced PAH by transferring miR-34a, miR-122, miR-124, and miR-127 (Ref. 218) and by shifting the balance from the ACE/AngII/AT1R axis toward ACE2/Ang(1–7)/Mas axis (Ref. 219). MSC-derived EVs also reduced monocrotaline-induced lung fibrosis, right ventricular hypertrophy, and pulmonary vascular remodelling by regulating the WNT5A/BMP signalling pathway (Ref. 220). In experimental hypoxia-induced PAH, macrophages were polarized to M2 phenotype by MSC-derived EVs, leading to reduced levels of inflammatory mediators and increased levels of IL-10, inhibiting over-proliferation of pulmonary artery smooth muscle cells (Ref. 221).

## Concluding remarks

Significant progress has been made in translational medicine for respiratory diseases; however, more precise biomarkers and effective therapies remain an urgent need. EVs have emerged as essential mediators of intercellular communication by interacting and transferring their content to recipient cells, thus regulating various biological pathways. Their ability to modulate resident and immune cell responses, inflammation, and tissue repair – particularly in respiratory diseases – highlights their multimodal potential. By enabling the identification of unique signatures that may distinguish pathological conditions and severity with high accuracy, EVs may hold an optimal role for diagnosis. In addition, MSC-derived EVs carry anti-inflammatory and regenerative factors that can influence cellular signalling processing, offering therapeutic avenues for lung inflammatory and fibrotic conditions. Lastly, EVs possess intrinsic properties that make them optimal candidates for gene/drug delivery by combining natural biocompatibility with the ability to target specific cells and tissues.

Despite encouraging pre-clinical findings, several challenges should be addressed before EVs become a tool in the clinical scenario. A major barrier is the need for high-quality, standardized methods for EV isolation and characterization. Current techniques frequently yield heterogeneous populations of EVs, which can hamper reproducibility and clinical development. Improved protocols for EV purification associated with rigorous characterization will be fundamental to ensuring consistency in therapeutic development. Moreover, the EV research field needs to adopt more transparent and stringent reporting practices in scientific publications to facilitate cross-study comparisons.

Although current evidence strongly supports the role of EVs in lung tissue repair and inflammation modulation, large-scale clinical studies are needed to establish standardized dosing, delivery methods, and long-term safety and efficacy. In parallel, interdisciplinary collaboration will be crucial in overcoming existing technical and regulatory barriers and in establishing guidelines for EV-based products to streamline their approval process.

In conclusion, EVs represent a transformative tool in biomedicine, with vast potential for both diagnostics and therapeutics. However, their successful translation into patient care will depend on overcoming current limitations in isolation techniques, biomarker specificity, and clinical validation. By addressing these challenges through rigorous research and collaborative efforts, the EV field can pave the way for novel, effective treatments for respiratory diseases and beyond. The future of EV-based medicine is promising, but its realization hinges on continued innovation, standardization, and evidence-based development.
